# Identification of New Features from Known Bacterial Protective Vaccine Antigens Enhances Rational Vaccine Design

**DOI:** 10.3389/fimmu.2017.01382

**Published:** 2017-10-26

**Authors:** Edison Ong, Mei U Wong, Yongqun He

**Affiliations:** ^1^Department of Computational Medicine and Bioinformatics, University of Michigan, Ann Arbor, MI, United States; ^2^Unit for Laboratory Animal Medicine, Department of Microbiology and Immunology, University of Michigan, Ann Arbor, MI, United States; ^3^Center of Computational Medicine and Bioinformatics, University of Michigan, Ann Arbor, MI, United States

**Keywords:** vaccine design, protective antigen, reverse vaccinology, adhesin probability, subcellular localization, conserved domains, transmembrane proteins, functional analysis

## Abstract

With many protective vaccine antigens reported in the literature and verified experimentally, how to use the knowledge mined from these antigens to support rational vaccine design and study underlying design mechanism remains unclear. In order to address the problem, a systematic bioinformatics analysis was performed on 291 Gram-positive and Gram-negative bacterial protective antigens with experimental evidence manually curated in the Protegen database. The bioinformatics analyses evaluated included subcellular localization, adhesin probability, peptide signaling, transmembrane α-helix and β-barrel, conserved domain, Clusters of Orthologous Groups, and Gene Ontology functional annotations. Here we showed the critical role of adhesins, along with subcellular localization, peptide signaling, in predicting secreted extracellular or surface-exposed protective antigens, with mechanistic explanations supported by functional analysis. We also found a significant negative correlation of transmembrane α-helix to antigen protectiveness in Gram-positive and Gram-negative pathogens, while a positive correlation of transmembrane β-barrel was observed in Gram-negative pathogens. The commonly less-focused cytoplasmic and cytoplasmic membrane proteins could be potentially predicted with the help of other selection criteria such as adhesin probability and functional analysis. The significant findings in this study can support rational vaccine design and enhance our understanding of vaccine design mechanisms.

## Introduction

Vaccination is considered as the most effective medical intervention ever introduced in modern medicine (1) and has prevented 103 million cases of infectious diseases in the United States since 1924 (2). However, it is still difficult to develop safe and effective vaccines against many infectious diseases including tuberculosis, HIV, and malaria (3). The emerging reverse vaccinology (RV) addresses the challenge through rational vaccine design by predicting vaccine antigen based on bioinformatics analysis of pathogen genomes (4, 5). The first application of RV in Group B *meningococcus* (MenB) vaccine development predicted 350 surface-exposed proteins from MenB, and the following experiments verified 25 of them capable of inducing bactericidal antibodies (6). This finding led to the approval of the first MenB vaccine, Bexsero, for use in the Europe (7), and United States (8). The success of Bexsero is a milestone for rational vaccine design and RV has also been applied in vaccine prediction against other challenging pathogens such as *Mycobacterium tuberculosis* (9).

Many selection criteria have been applied to vaccine antigen prediction, but a deep understanding of the rationale behind their usage is still missing. The initial RV study of MenB vaccine prediction used the subcellular localization (SCL) as a major selection criterion, given that the humoral immunity is vital to host protection against MenB and the protective antigens (PAgs) inducing antibody response are primarily located in extracellular or outer membrane (6). However, the preference of vaccine antigens in specific SCL varies across different pathogens, and SCL might not be equivalently critical for those pathogens against which cell-mediated immunity plays a major role. Another frequently used criterion is the number of transmembrane α-helices (TMHs) due to the difficulty in the isolation of proteins with more than one TMH (10). Nonetheless, it is unclear whether the number of TMH, and possibly transmembrane β-barrel (TMB), of a protein correlates with vaccine protection. Adhesin is crucial to pathogen invasion into host cells (11) but the usage of adhesin probability (AP) has not been widely appreciated. Other criteria including signal peptides, conserved domains, and biological function analysis (10) have been used in different RV tools [e.g., NERVE (12), Vaxign (13), and Jenner-predict server (14)], and machine-learning techniques are also applied to vaccine design studies (15, 16). However, the significance and association of above criteria with the protectiveness of bacterial PAgs is still lacking. The identification of such association is essential to improve vaccine antigen prediction and design studies.

The goal of this study is to systematically analyze known bacterial PAgs reported in the literature and identify underlying design mechanisms for better rational vaccine prediction. Our study uses PAgs collected from Protegen with antigen information and experimental protection evidence manually annotated from peer-reviewed articles (17). The significance and association of these Protegen PAgs are analyzed using bioinformatics tools for SLC (18), AP (19), signal peptide (20), TMH (21) and TMB (22), conserved domains (23), Clusters of Orthologous Groups (COG) (24), and gene ontology (GO) (25). This report provides a systematic analysis of protein properties and biological functions associated with known bacterial PAgs in the interest of supporting future rational vaccine prediction and design.

## Materials and Methods

### Protective Antigens and Background Pan-Proteome Non-Protective Protein Sequences

Protective antigens in G^+^ and G^−^ bacteria with supporting experimental evidence were downloaded from Protegen database (Table S1 in Supplementary Material). The most common experimental evidence is the protection results against virulent bacterial challenge in laboratory animal models. Reported assay results that correlate to protection or immune responses are also considered. Using the Gram-positive (G^+^) and Gram-negative pathogen information provided along with the PAgs from Protegen, all protein-coding sequences of these pathogens were downloaded from the UniProt database (26). The taxonomy IDs reported in Protegen were queried against UniProt for possible pan-proteome sequences. The detail of taxonomy ID mapping between the reported G^+^ and G^−^ pathogens from Protegen and their corresponding pan-proteome in UniProt is available in Table S2 in Supplementary Material. By merging all the pan-proteome protein sequences from UniProt, we obtained the background proteome for two groups used in this study: G^+^ and G^−^ pathogen background proteomes. There is no curated dataset of non-protective G^+^ and G^−^ proteins available in the literature. The non-protective protein datasets were generated by applying similar strategies reported in previous vaccine design studies (15, 27, 28). Specifically, the G^+^ and G^−^ pan-proteomes downloaded from UniProt were first aligned to Protegen PAg sequences using BLAST (29). Then sequences that shared similar homology with the Protegen PAgs (*E*-value ≤10 and have a shared percent identity of 10%) were removed from the datasets. All the remaining sequences within the datasets were considered as non-protective proteins throughout the entire study. The non-protective proteins generated in this study only provide an estimated survey of the true non-protective datasets and some non-protective proteins included in this study could have never been tested for the protective capacity.

### Protein Property Computations

In this paper, five types of protein properties were computed: (i) SCL, (ii) AP, (iii) signal peptide, (iv) TMH, and (v) TMB (Table S8 in Supplementary Material).

For SCL computation, all sequences were computed for tentative SCL locations by running through PSORTb v3.0 program (18). Briefly, PSORTb uses Bayesian network to integrate different SCL location prediction modules such as support vector machine, SCL-BLAST, and motif-based modules. The program predicts and assigns score for each possible SLC locations of the input sequence, and the location with the highest score is returned. In this study, the default setting was used besides specifying the G^+^ or G^−^ of input sequences.

The AP of all sequences was computed using SPAAN program with default setting (19). SPAAN calculates probability of being adhesin for an input sequence using neural network with five features including amino-acid frequencies, multiplet frequencies, dipeptide frequencies, charge composition, and hydrophobic composition. Sachdeva et al. reported 89% sensitivity and 100% specificity when the cutoff value AP ≥ 0.51 was used (19), and therefore the same threshold was applied in this study.

Prediction of signal protein secretion of all sequences was calculated by SignalP 4.1 standalone version (20), which is built solely on neural network to discriminate signal peptides from transmembrane regions. The discrimination score (D-score) computed by SignalP provides a value for protein secretion. As suggested by SignalP^1^, the threshold value of D-score of 0.45 for G^+^ and 0.51 for G^−^ provides the best sensitivity in signal peptide detection. In this study, the suggested cutoff values were used and the default configuration was applied besides specifying the G^+^ or G^−^ of input sequences.

The TMH was computed using TMHMM 2.0 (21) with default settings and the number of TMH of the input G^+^ and G^−^ pathogen sequences was reported. In brief, the tool uses hidden Markov model to predict transmembrane state of the input sequences and the Krogh et al. reported 97–98% prediction sensitivity (21).

The TMB was computed using PROFtmb tool, which is also a hidden Markov model-based prediction program (22). Only TMB of G^−^ pathogen sequences were computed because classical G^+^ bacteria do not contain β-barrel membrane proteins (30). Based on the performance evaluation of the PROFtmb on discriminating transmembrane vs. non-transmembrane β-barrel using the whole protein dataset by Bigelow et al. (22), a cutoff value of ≥0.6 accuracy was chosen in order to achieve a balance with coverage.

### Protein Sequence Property Computations

The PAg sequences, non-protective protein and background proteome sequences were functionally annotated with (i) Pfam conserved domains, (ii) COG functional classifications, and (iii) GO biological process (BP), molecular function (MF) and cellular component (CC) terms (Table S8 in Supplementary Material).

The PfamScan tool was used to annotate the conserved domains in all PAg, non-protective proteins and background proteomes. The sequences were aligned using the downloaded Pfam-A domain hidden Markov models (23).

The sequences of all PAgs were scanned for COG clusters using HMMER^2^ with the hidden Markov models downloaded from the EggNog 4.5 database (31). Each input sequence was initially assigned with one ENOG identifier, which was then mapped to the corresponding COG cluster. For background proteomes and non-protective proteins, the COG cluster identifiers were retrieved directly from the UniProt database.

The PAg sequences were submitted to Argot2 web server for GO annotation prediction (32). The GO information of non-protective proteins and background proteomes were directly downloaded from UniProt database.

### Statistical Analysis

Unless specified, the statistical significance of the association between reported PAgs and computed protein properties including SCL, AP, signal peptide, TMH, and TMB were calculated using one-way Fisher’s exact test since we were only interested in over-representation of properties in PAgs only. For the *ad hoc* analysis of specific property (e.g., SCL prediction), the significance of individual sub-property (e.g., individual SCL locations such as extracellular, cell wall, cytoplasmic membrane, and cytoplasm in G^+^ bacteria) were further examined by performing one vs. other Fisher’s exact test and the resulting *p*-value was adjusted by applying Bonferroni correction.

The over-representation of conserved domains, COG clusters, and GO BP, MF, CC terms among Protegen PAgs were tested using Fisher’s exact test and adjusted using Benjamini–Hochberg–Yekutieli procedure. In addition, the significant (adjusted *p* ≤ 0.05) GO terms (BP, MF, CC) were visualized in hierarchical format using GOfox (33). GOfox^3^ laid out GO terms using the internal hierarchical GO structure simplification algorithm since GO enrichment analysis tends to generate a large list of enriched GO terms (33).

## Results

Three sets of data were collected and generated for the bioinformatics analysis. Our study specifically analyzed frequently used PAg prediction features, including SCL, AP, signal peptide, TMH and TMB, conserved domain, and biological function analysis.

### Collection of Protective Vaccine Antigens, Background and Non-Protective Proteins

After removal of identical sequences, the curated Protegen dataset contained 81 and 210 non-redundant vaccine PAgs from 14 Gram-positive (G^+^) and 34 Gram-negative (G^−^) bacteria, respectively (Table S1 in Supplementary Material). The corresponding pan-proteomes of these G^+^ and G^−^ pathogens were downloaded from the UniProt database (26) as the background proteomes, which included 39,397 G^+^ and 73,371 G^−^ peptide sequences (Table S2 in Supplementary Material). A set of non-protective proteins were selected from background proteome as described in Materials and Methods and other RV studies (15, 16, 27, 28), and contained 4,954 G^+^ and 5,478 G^−^ pathogen peptide sequences.

### Subcellular Localization Analysis

**Figure 1:**
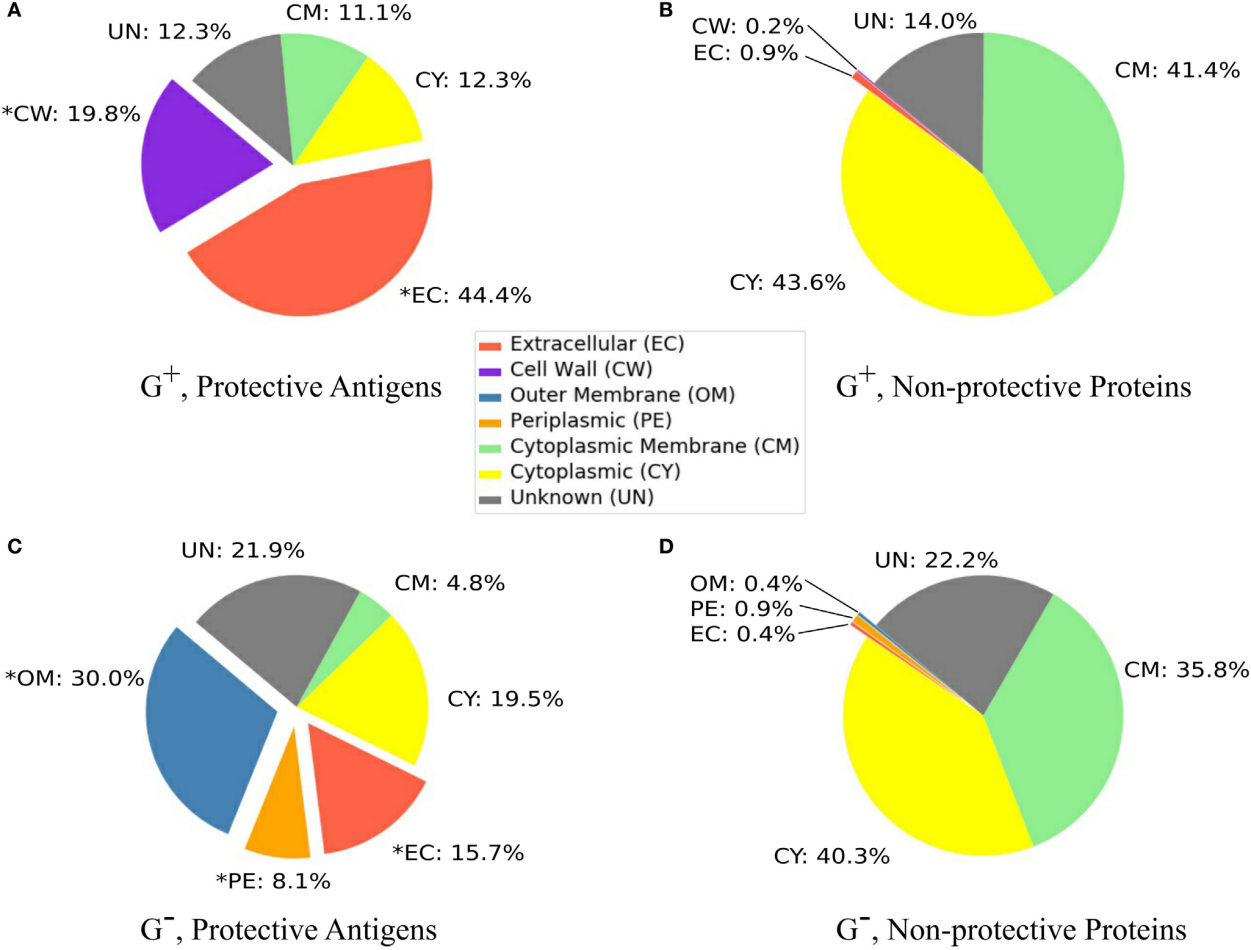
Subcellular localization profiles. G^+^ and G^−^ bacterial protective antigens showed significantly higher (*p* < 0.01) proportions of EC (G^+^ and G^−^), CW (G^+^), and PE and OM (G^−^) **(A,C)** compared with the non-protective proteins **(B,D)** (abbreviations shown in the middle color key legend). Only PAgs and non-protective proteins are displayed and the composition of background proteomes and non-protective proteins are very alike (Figure S1 in Supplementary Material). The significant over-representation of PAgs’ subcellular localization prediction compared with non-protective proteins is indicated with “*” (*p* < 0.01).

Our analysis found that 44.4% and 19.8% of PAgs in G^+^ bacteria located in extracellular space and cell wall, respectively (Figure 1A). In comparison, only 1.7% and 1.2% of the G^+^ non-protective proteins were extracellular and cell wall proteins, respectively (Figure 1B). Our statistical analysis showed significant over-representation of PAgs in these two SCLs (*p* < 0.01). In G^−^ bacteria, 15.7%, 30.0%, and 8.1% of PAgs were extracellular, outer membrane, and periplasmic proteins, respectively (Figure 1C). Compared with the corresponding SCL proportions in G^−^ non-protective proteins (0.4, 0.4, and 0.9%) (Figure 1D), these three locations were significantly over-represented in PAgs (*p* < 0.01). In non-protective proteins, most proteins (78.3% in G^+^ and 67.7% in G^−^) were localized in the cytoplasmic or cytoplasmic membrane (Figures 1B,D) but these two SCL locations also accounted for 26.8% G^+^ and 31.1% G^−^ of the reported PAgs (Figures 1A,C). The SCL predictions of background proteome were shown in Figure S1 in Supplementary Material.

To confirm the SCL analysis results, we also analyzed signal peptides using SignalP (20), which predicted the presence of signal sequences of the majority of synthesized proteins designated to secretory pathways. The distribution histograms of the calculated score for PAgs, non-protective proteins, and background proteomes were plotted (Figure S2 in Supplementary Material). The signal peptide scores of extracellular (both G^+^ and G^−^) or surface-exposed proteins (cell wall for G^+^ and outer membrane for G^−^) showed that a large fraction of PAgs was predicted to be secreted signal peptides (Figure S2 and Table S3 in Supplementary Material).

### Adhesin Probability Analysis

**Figure 2:**
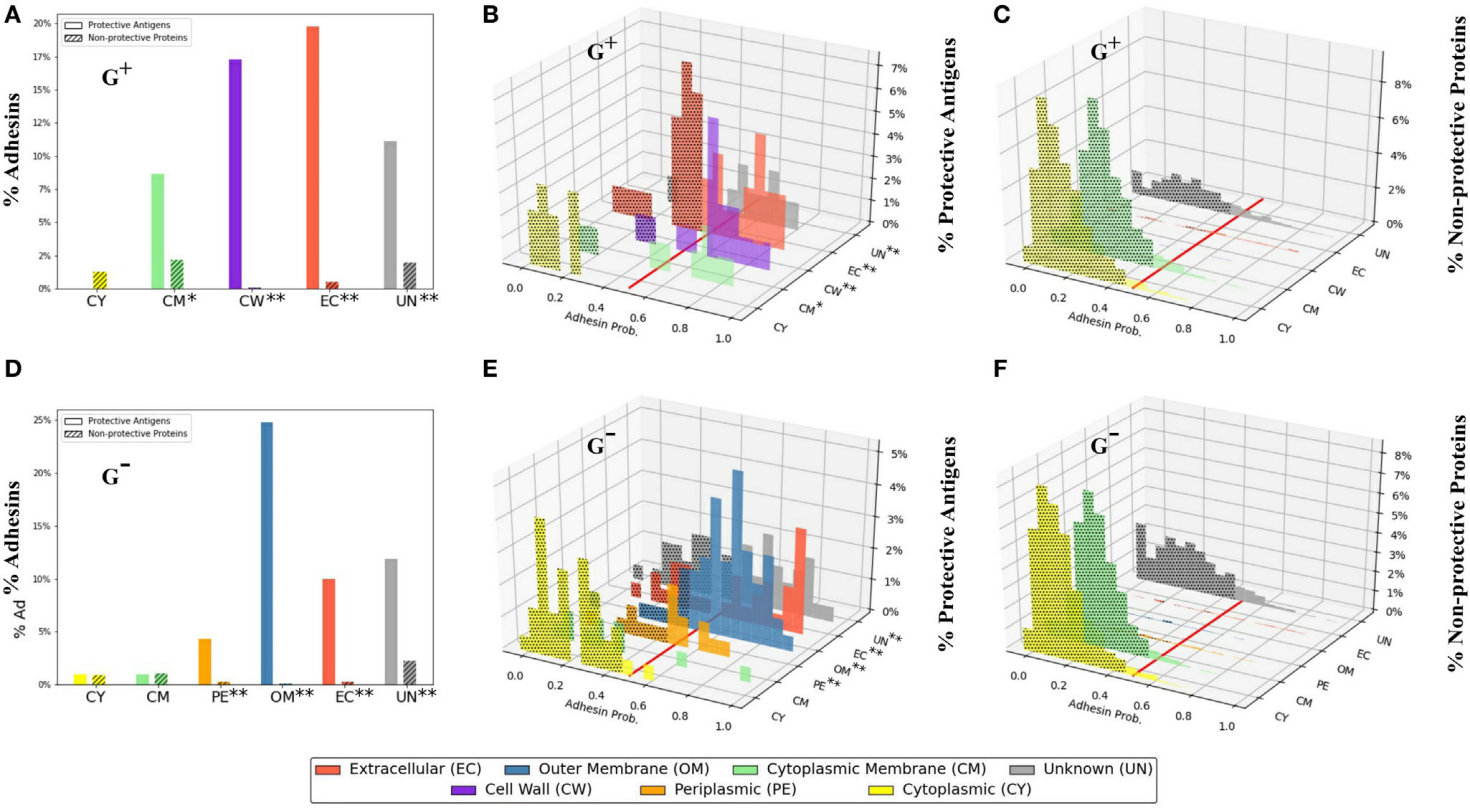
Profiles of adhesin probabilities of protective antigens (PAgs) and non-protective proteins with different subcellular localizations. The top three panels **(A–C)** show G^+^ pathogens, and the bottom three panels show G^−^ pathogens. Specifically, the first column **(A,D)** represents the overall percentages of adhesin probabilities. The second column (**B,E**) and third column **(C,F)** show adhesin probability distributions of PAg and non-protective proteins, respectively. The red line in **(B,C,E,F)** indicates adhesin probability cutoff of no <0.51. Overall, PAgs have significantly higher (*p* < 0.01) percentages in EC (G^+^ and G^−^), CW (G^+^), and PE and OM (G^−^) (abbreviations shown in bottom color key legend). Interestingly, CM in G^+^ is also significant (*p* < 0.05) when coupled with adhesin probability. See the text for detailed discussion. The significant over-representation of PAgs’ adhesin probabilities at different subcellular localizations compared with non-protective proteins is indicated with “*” (*p* < 0.05) or “**” (*p* < 0.01).

Adhesins are proteins critical for bacterial pathogens to invade host cells and cause infections (11). Over half of the PAgs could be identified with AP (56.8% of G^+^ and 52.8% of G^−^) using the suggested cutoff of no <0.51 (19). The AP of proteins with different SCLs also had different patterns (Figure 2). Specifically, comparing PAgs (Figures 2B,E) and non-protective proteins (Figures 2C,F), PAgs with SCL locations other than cytoplasmic membrane and cytoplasm generally showed increasing trend in AP. There were 87.5% G^+^ PAgs in the cell wall and 82.5% G^−^ PAgs in outer membrane that were also adhesins, compared with 37.5% G^+^ and 20% G^−^ non-protective proteins in the cell wall and outer membrane, respectively (Figure 2; Table S4 in Supplementary Material). This high preference of surface-exposed proteins (cell wall for G^+^ and outer membrane for G^−^) with high AP was significant (*p* < 0.01, Figure 2) and illustrated the importance of SCL and AP as two major criteria in vaccine design. Additionally, 90.0% and 54.3% of the PAgs in G^+^ and G^−^ bacteria with unknown SCL were in fact predicted to be adhesins. Therefore, utilizing AP with SCL could potentially overcome the limitation of excluding “Unknown” SCL and avoid inaccuracy generated by individual SCL prediction tool. For PAgs located at the cytoplasmic membrane and cytoplasm, the computed AP also showed different patterns between G^+^ and G^−^ (Figures 2B,E). G^+^ PAgs in cytoplasmic membrane were more likely adhesins (77.8%), while in G^−^ only 20.0% were adhesins (Table S4 in Supplementary Material). For cytoplasm, PAgs were both unlikely adhesins (0% for G^+^ and 4.9% for G^−^, Table S4 in Supplementary Material). AP prediction of background proteome is also shown in Figure S3 in Supplementary Material.

### Transmembrane α-Helix and β-Barrel

**Figure 3:**
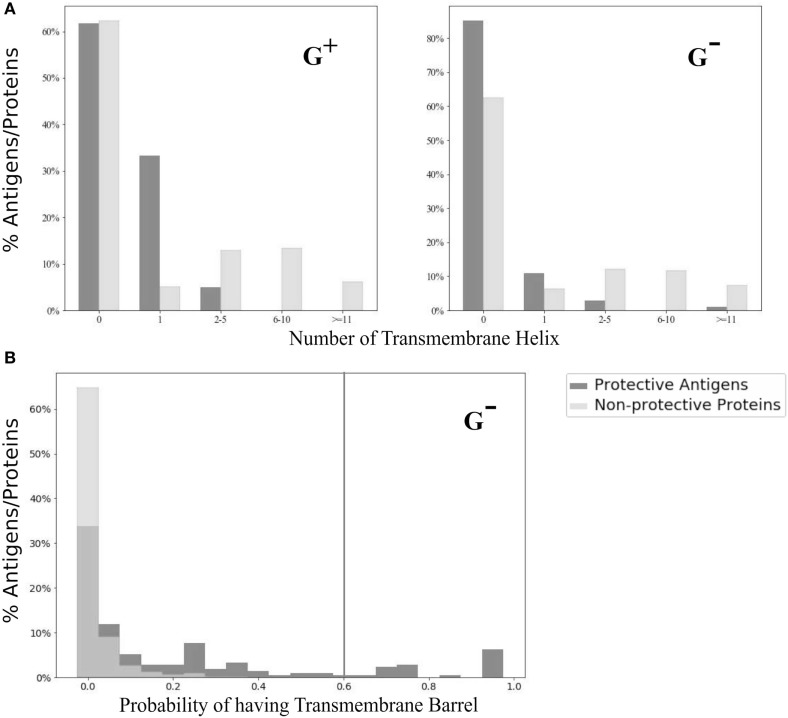
Transmembrane α-helix (G^+^ and G^−^) and β-barrel (G^−^ only) profiles. As compared with non-protective proteins, there were much higher percentages of protective antigens with zero or one transmembrane α-helix **(A)**. For transmembrane β-barrel **(B)**, only two (0.0004%) out of all non-protective proteins had probability higher than the designated cutoff (indicated as black vertical line) described in method.

We analyzed and compared the TMH profiles between PAgs and non-protective proteins. Specifically, none of the PAgs located at the cell wall (G^+^), outer membrane, or periplasm (G^−^) had more than one TMH (Figure 3A). There were two G^−^ PAgs with more than 10 TMH (lipoprotein signal peptidases in *Brucella melitensis* and l-lactate permease in *Neisseria meningitides*). The β-barrel analysis was only performed for G^−^ pathogens because classical G^+^ bacteria do not contain β-barrel membrane proteins (30). Using the probability cutoff of 0.60, our study found that 12.9% of Gram-negative PAgs predicted to have TMB compared with <0.001% in non-protective proteins (Figure 3B).

### Conserved Domain Analysis

Conserved domains represent functional units in proteins and some domains are more frequently associated with PAgs (14, 34). Our analysis identified eight conserved domains that were only frequently found among reported PAgs (Table 1). These domains included “autotransporter β-domain,” “outer membrane protein β-barrel domain,” “fimbrial protein,” “TonB-dependent receptor plug domains,” “OmpH-like outer membrane protein,” and “extended signal peptide of type V secretion system.” The full list of all predicted conserved domains and their frequencies in PAgs and non-protective proteins can be found in Table S5 in Supplementary Material.

**Table 1:** Frequent Pfam-A conserved domains among reported PAgs.

Pfam domain description	Protective antigen count
Autotransporter β-domain	11
Outer membrane protein β-barrel domain	10
Fimbrial protein	10
ATPase family associated with various cellular activities (AAA)	9
TonB-dependent receptor plug domain	8
Outer membrane protein (OmpH-like)	5
ABC transporter	5
Extended signal peptide of Type V secretion system	5

*The most over-represented (PAg count ≥5) Pfam-A conserved domains among reported PAgs were listed. The top two frequently found Pfam-A conserved domains among reported PAgs were β-barrel domains, which support the positive selection of transmembrane β-barrel in PAg prediction. In addition to β-barrel domains, proteins with over-represented conserved domains were more likely related to the pathogenesis of bacteria including pathogen colonization and invasion, and therefore could be used as a good indicator of PAg prediction*.

### Functional Analysis

**Figure 4:**
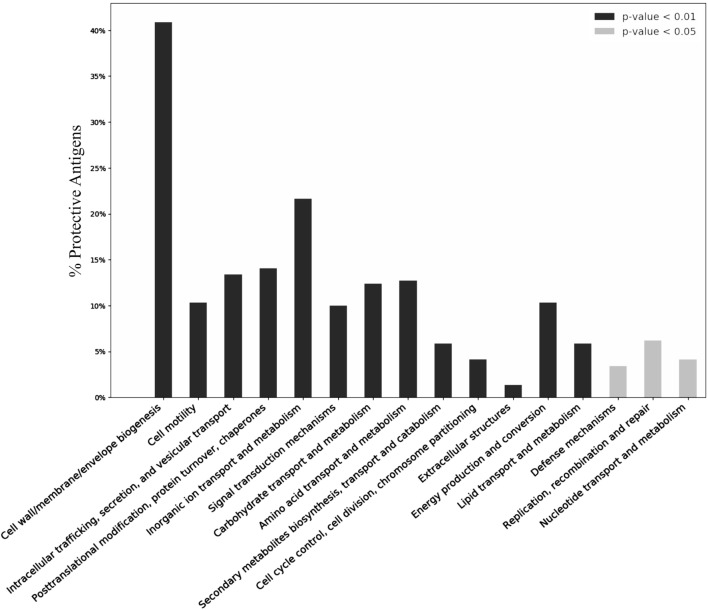
Over-representation of COG clustering profiles among reported protective antigens (PAgs). Over 40% of the reported PAgs belong to the cluster “Cell wall/membrane/envelop biogenesis,” which agrees with common knowledge of using surface-exposed proteins as a key criterion in vaccine antigen prediction. Other COG clusters related to pathogen motility, secretion, signal transduction, and transportation are also significantly enriched in PAgs as compared with non-protective proteins. See the text for detailed discussion. The significant over-representation of PAgs’ COG clusters compared with non-protective proteins is colored with gray (*p* < 0.05) and black (*p* < 0.01).

The functional annotations were analyzed using the COG and GO. COG includes 26 functional clusters (24). Our COG analysis of PAgs identified 16 COG functional categories that were significantly enriched (adjusted *p* < 0.05) in PAgs (Figure 4; Table S6 in Supplementary Material). Four COG clusters “cell wall/membrane envelope biogenesis,” “cell motility,” “signal transduction mechanisms,” and “extracellular structures” were notably enriched in PAgs.

**Figure 5:**
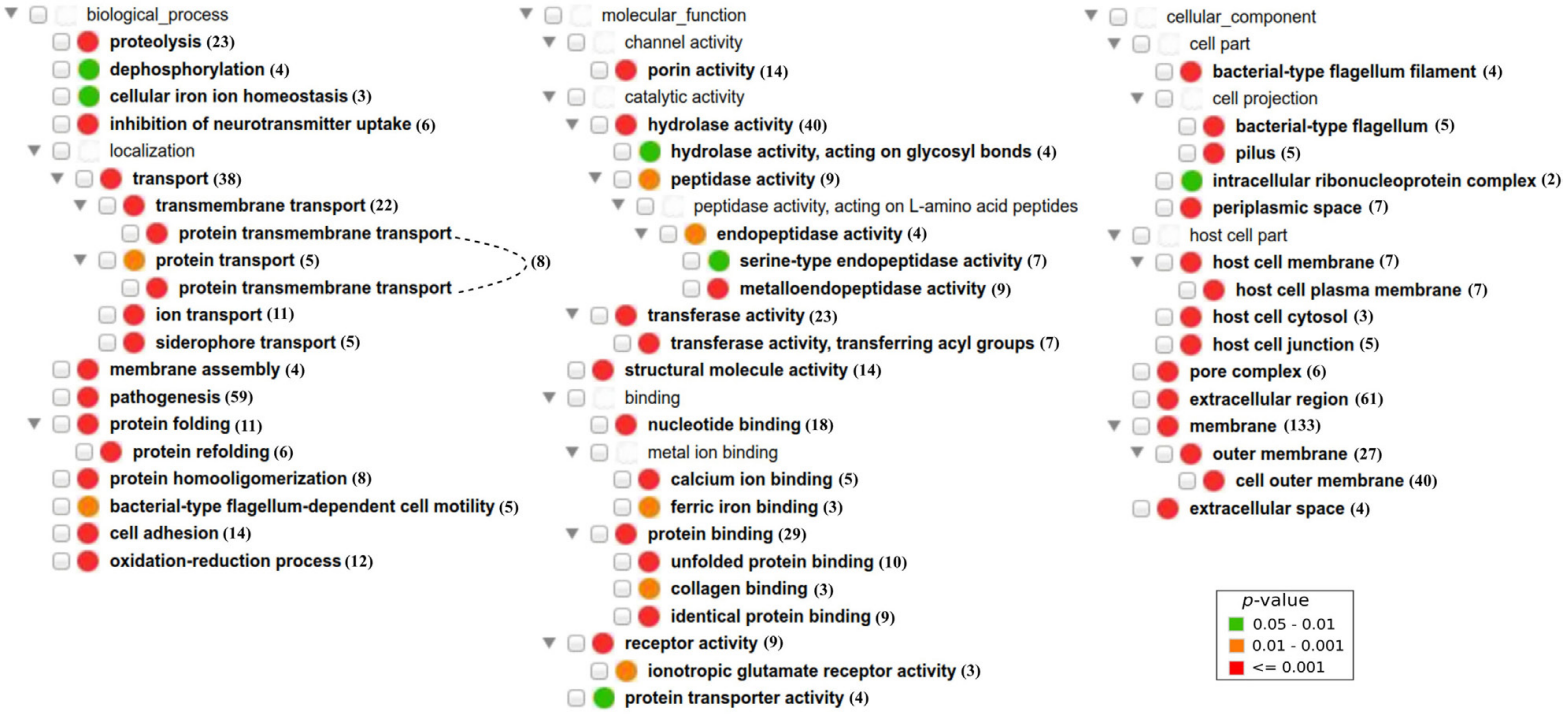
Over-representation of GO BP, MF, and CC term profiles among reported protective antigens (PAgs) and visualized using the GOfox tool. The number next to each GO term indicates the number of PAg with the corresponding GO functional annotation. Similar to COG clustering, GO terms that are related to pathogen motility, secretion, signal transduction, and transportation are also significantly enriched in PAgs as compared with non-protective proteins. The GO CC terms also supported the high preference of extracellular, surface-exposed (cell wall in G^+^ and outer membrane in G^−^), and periplasmic (G^−^) PAgs. The significant over-representation of PAgs’ GO terms compared with non-protective proteins is color-coded following the legend in the lower right corner.

We also analyzed enriched GO terms from the three GO branches: biological process (BP), molecular function (MF), and cellular component (CC) (25). Eighteen GO BP terms were found significantly enriched (adjusted *p* < 0.05) in bacterial PAgs, including “pathogenesis” as the most significantly enriched term among PAgs in bacterial pathogens (Figure 5; Table S7 in Supplementary Material). BPs related to pathogen invasion (e.g., “cell adhesion” and “proteolysis”) and terms related to transporter (e.g., “transmembrane transport”) were significantly over-represented among PAgs. Twenty GO MF terms were significantly enriched (adjusted *p* < 0.05), including those related to invasion (e.g., “peptidase activity”) and transportation (e.g., “transferase activity” and “receptor activity”). Fifteen GO CC terms were significantly enriched (adjusted *p* < 0.05). In agreement with the SCL prediction results, extracellular or surface-exposed CC terms were significantly over-represented among reported PAgs. In addition, CC terms that were related to bacterial colonization and invasion within host such as “bacterial-type flagellum filament,” “pilus,” “host cell part,” “host cell plasma membrane,” and “host cell junction” were also enriched, suggesting PAgs’ role in the interactions between bacteria and the host cells.

## Discussion

Although extensive research has been conducted, modern vaccine research and development still faces challenges of quick and accurate development of vaccines in response to major infectious diseases [e.g., tuberculosis (3)], outbreaks [e.g., Ebola and Zika virus (35, 36)], and new drug-resistant pathogens (37). Our efforts to develop vaccines using traditional methods have not been successful to address these challenges. The future success of effective vaccine development relies on powerful rational vaccine design including reverse and structural vaccinology (1) and based on our deeper understanding of vaccination mechanism. By systematically studying and comparing bacterial PAgs and non-protective proteins, our comprehensive bioinformatics study analyzed key criteria for vaccine design including various protein properties and biological functions. The summarized characteristics in this study are specifically used for bacterial model PAg prediction and might not hold true for viral or parasitic pathogens. The results of this study confirmed and provided details on the usage of these prediction criteria, including SCL, AP, signal peptides, TMH and TMB, conserved domains, and biological function annotations, for RV prediction against bacterial pathogen. Most importantly, our results suggested new insights toward rational vaccine prediction and design.

In accordance with secreted extracellular or surface-exposed antigens commonly known to be PAgs, our study observed the differences among the SCL profiles of G^+^ and G^−^ bacterial PAgs. In terms of extracellular proteins, G^+^ bacterial PAgs had a much higher percentage (44%) being PAgs than G^−^ bacterial PAgs (15.7%). We also found a strong correlation between the presence of secretory signal peptides and PAgs. Approximately half of the PAgs (over 45% in both G^+^ and G^−^) were predicted to be signal peptides (Table S3 in Supplementary Material; Figure 4). Coupling the selection of SCL and signal peptides, particularly in G^+^ bacterial pathogens, pose a viable option for a more precise PAg prediction. On the other hand, 19.8% cell wall proteins in G^+^ and 30.0% outer membrane proteins in G^−^ bacteria were surface-exposed PAgs (Figures 1A,C). The G^+^ bacterial PAgs showed higher preference in extracellular proteins, while both G^+^ and G^−^ bacterial PAgs shared similar proportions as surface-exposed proteins.

Moreover, 8.1% G^−^ PAgs were in the periplasm, a subcellular location that vaccine researchers often ignore due to lack of direct interaction with the host immune cells. Hence, the percentage of periplasmic PAgs was significant (*p* < 0.05, Figure 1C) and over-represented (Figure 5) when taking the non-protective periplasmic proteins (0.9%) into consideration. It is possible that G^−^ bacterial periplasmic proteins can be released extracellularly after being packed within outer membrane vesicles and can induce strong immune responses (38, 39). These periplasmic proteins can be potentially a good source of PAg candidates when coupling with other selection criteria such as functional analysis.

The results of our study highlight the importance of AP and its effect in improving RV prediction when combined with SCL. Adhesin is critical for bacterial invasion and is capable of inducing strong immune responses (11). Adhesins can also function as enzymes and mediate a main part of bacterial pathogenesis (40). The majority of vaccine design studies do not incorporate AP in their selection pipeline (6, 15, 16, 27), and AP as a selection criterion is currently underused and poorly investigated in the vaccine development field. Our study managed to identify over 50% of the PAgs with AP as the only criterion. The prediction of coupling SCL and AP was even more significant, with the identification of over 80% cell wall (G^+^) and outer membrane (G^−^) PAgs (Table S4 in Supplementary Material). By addressing the importance of adhesin playing an important role in vaccine development, we hope to promote the AP as a viable option in future vaccine design studies.

The functional analysis of adhesive PAgs in our study proposes a mechanistic explanation of their roles in pathogen colonization and invasion. Cell motility is one of the most important steps in host colonization and invasion, and the bacterial movement requires structure such as flagellum and pillus for cell adhesion and colonization (41), and cell motility related COG clusters and GO terms were significantly enriched (Figures 4 and 5). Pilli are composed of fimbrial and other proteins (41), and the Pfam domain “fimbrial protein” was highly conserved among the reported PAgs (Table 1). GO BP term “proteolysis” and GO MF terms “peptidase activity” (Figure 5) were also found to be significant in the functional analysis. For instance, *Yersinia pestis* can produce the surface protease to mediate invasion into host endothelial cells (42). The pili, fimbri, and protease mentioned earlier can occur as one of the various architectures of adhesins (40). Given these important roles of adhesins, more investigations of adhesins as potential PAgs and how they induce protective immunity are much deserved.

Our study showed two distinct correlation patterns of the PAgs protectiveness to the TMH and TMB. The TMH is more abundant in cytoplasmic or inner membranes, and the TMB type is more likely located in bacterial outer membranes (43). Our study confirmed that TMH proteins with more than one TMH were not typically used for vaccine development (10) (Figure 3A; Figure S5 in Supplementary Material). The two exceptional proteins that had more than 10 TMHs, which were *Brucella* lipoprotein signal peptidase and *Neisseria meningitides* l-lactate permease. *Brucella* lipoprotein signal peptidase is a known virulence factor, which is involved in lipopolysaccharides biosynthesis (44). The *N. meningitides* l-lactate permease is a protein required by *N. meningitides* during bacteraemic infection and induces protective immunity in systemic *meningococcal* infection (45). Different from TMH, our study indicated that the presence of TMB was associated with significantly higher portions (*p* < 0.01 from χ^2^ test) of G^−^ PAgs (Figure 3B). In particular, none of the G^−^ outer membrane non-protective proteins was predicted to have TMB. Our results suggested the use of TMH as a negative and TMB as a positive selection criterion in future vaccine development.

Although not usually considered as PAgs, large portions (26.8% G^+^ and 31.1% G^−^) of cytoplasmic and cytoplasmic membrane proteins were found to be PAgs (Figures 1A,C). Compared with a much larger size of cytoplasmic and cytoplasmic membrane non-protective proteins, this fraction of PAgs was not significant. However, the ignorance of proteins located at these two SCLs might hinder the productivity of effective PAg prediction. Cytoplasmic and cytoplasmic membrane proteins might not induce humoral immune response due to their SCLs, but these proteins often time can be potent inducers of cell-mediated immunity. For example, the cytoplasmic catalase-peroxidase protein in *M. tuberculosis*, which contributes to intracellular survival within host macrophage by protecting against reactive oxygen species (46), is able to induce protective immunity (47). How to accurately predict cytoplasmic PAgs remains a big challenge but it can be potentially addressed using multiple features such as AP, conserved domains, COG clusters, and GO terms. Particularly in G^+^ cytoplasmic membrane, PAgs showed significant over-representation (*p* < 0.05) when coupled with AP prediction. Conserved domains have been reported as a viable option in the PAg prediction (14). In our study, many conserved domains were frequently found among PAgs and each domain might link to important bacterial biological functions within the host such as “TonB-dependent receptor plug domain.” As a strategy in antibiotics resistance is the bacterial efflux pumps (48), TonB-dependent receptor is a G^−^ bacterial protein responsible for the transportation of large ion complex and has been identified as potent vaccine PAgs (49). The over-represented COG clusters and GO terms among the reported PAgs suggested a viable alternative to overcome the challenge of identifying cytoplasmic and cytoplasmic membrane PAgs and complement to current vaccine prediction studies.

The findings in this study can be translated into a predictive framework with different approaches to improve existing methods and achieve better identification and validation of novel PAgs. Even though traditional rule-based prediction has been successful in multiple studies (6, 9) and also applied in many tools (12–14), this type of “all-or-nothing” selection might fail to capture the relationship among different criteria (16). For example, a potential cytoplasmic or cytoplasmic membrane PAg would be immediately discarded from a study that includes surface-exposing SCL as one of the criterion. As indicated in our findings, the cytoplasmic or cytoplasmic membrane PAg could be predicted by incorporating other criteria such as AP, conserved domains, and biological functions. As a natural solution, a combinatory strategy has been proposed that assigns each criterion with a weight and synthesizes multiple criteria in a composite way such as weighted metrics (50). Candidate proteins that have low score in a set of rules could still achieve a reasonable score and are compensated by another set of selection criteria. Another advance technique is to apply machine-learning methods such as support vector machine, random forest, and neural network as described in many previous studies (15, 16, 28, 34). Even though the machine-learning-based prediction can overcome the “all-or-nothing” scenario, these methods have not captured all the significant features as reported in this study. For example, AP and conserved domains are not implemented in current ML-based prediction (15, 16, 28) except the preliminary study by Xiang and He (34), and none of these studies incorporated TMB and biological functional analysis into their prediction pipeline. The additional features given from our findings showed promising improvement on current machine-learning methods.

Based on the new discoveries reported in this study, we plan to explore the possibility of integrating these significant criteria along with other including MHC-epitope binding and structure on protein selection as vaccine candidates to improve our Vaxign software program (13). Even though our analysis focused on bacterial model, some criteria such as AP, signal peptide, transmembrane proteins, and pathogenesis-related conserved domains and biological functions can be extended to viral or parasitic PAgs prediction after further verification and analysis. The better understanding of the association between individual criterion and PAgs, as well as the inter-relation among different criteria, will provide new opportunities for more accurate and rational vaccine design, leading to better prevention and control of various infectious diseases.

## Author Contributions

EO and YH conceived and designed the study. EO and MW collected protective antigens and background proteome sequences. EO performed bioinformatics analyses of the protein properties and functional annotations. EO, MW, and YH prepared the manuscript. All authors participated in result interpretation, paper editing, discussion, and approved the paper publication.

## Conflict of Interest Statement

The authors declare that the research was conducted in the absence of any commercial or financial relationships that could be construed as a potential conflict of interest. The reviewer SZ and handling Editor declared their shared affiliation.

## Footnotes

^1^http://www.cbs.dtu.dk/services/SignalP/performance.php.

^2^http://hmmer.org/.

^3^http://gofox.hegroup.org/.
