# Supplementary material for: Identification of New Features from Known Bacterial Protective Vaccine Antigens Enhances Rational Vaccine Design
Source: Front Immunol. 2017 Oct 26;8:1382. doi: 10.3389/fimmu.2017.01382 (PMC5662880; doi:10.3389/fimmu.2017.01382)
Supplement: Supplementary file 1 [file Data_Sheet_1.docx]

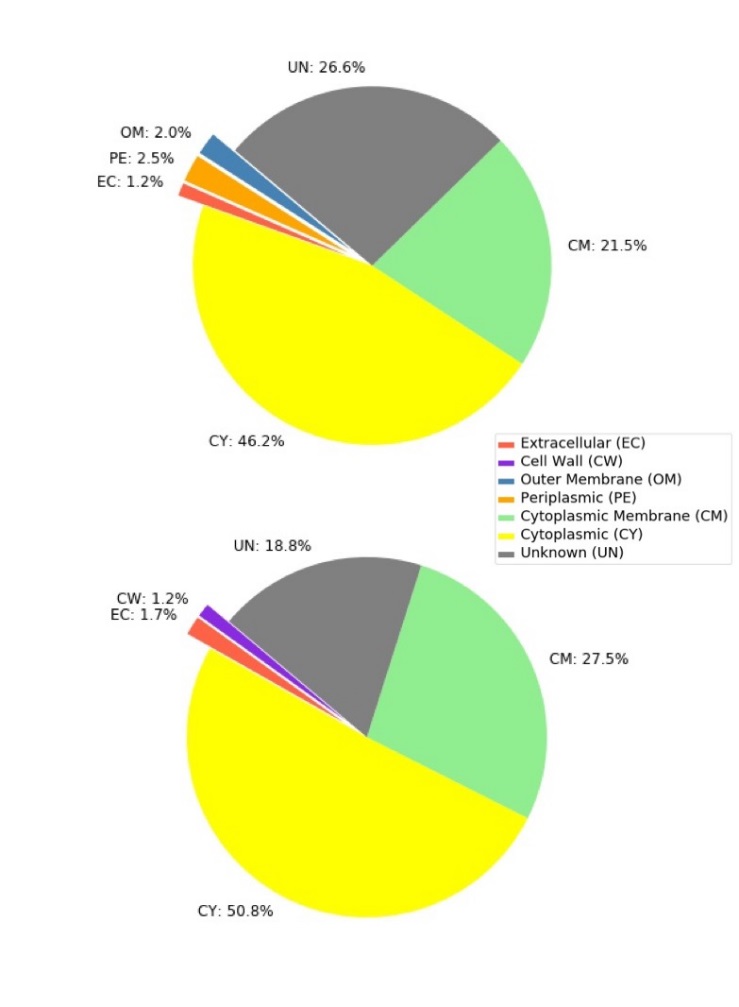


**Supplemental Figure S1.** Subcellular localization profile of background proteome.

**Supplemental Figure S2.** SignalP D-score distribution of protective antigens (a)&(d), non-protective proteins (b)&(e), and background proteome (c)&(e). Protective antigens shows significantly more signaling peptide predictions in subcellular locations including cellwall (Gram-positive), outer membrane and Periplasmic (Gram-negative), extracellular and unknown (both Grams).

**Supplemental Figure S3.** Adhesin probability profiles of protective antigens and background proteome with different subcellular localizations. The top three subfigures (a)-(c) are for Gram-positive, and the bottom three (d)-(f) are for Gram-negative bacteria. (a) and (d) represent the overall percentages of adhesins (SPAAN adhesin probability >= 0.51) in protective antigens and background proteome. The SPAAN overall adhesin probability distributions of protective antigens (b),(e), and background proteome (c),(f), are shown. Overall, the patterns of the comparison of protective antigens against non-protective proteins or background proteome are identical (see Figure 2).

**Supplemental Figure S4.** Transmembrane histograms of protective antigens (a)&(d), non-protective proteins (b)&(e), and background proteome (c)&(e). Protective antigens in all subcellular localization, except cytoplasmic in Gram-negative bacteria) shows less transmembrane helices (<=1). In Gram-negative bacteria, there are only two protective antigens in cytoplasm with over 5 transmembrane helices. Detail explanation of these two protective antigens are available in discussion.

**Supplemental Table S1.** All protective antigens of Gram-positive and Gram-negative bacteria and related information downloaded from Protegen database.

**(See attached Excel file)**

**Supplemental Table S2.** A list of Gram-positive and Gram-negative bacteria used in this study. The uniprot pan-proteomoe IDs are also included, along with the number protective antigens and background proteins count.

**(See attached Excel file)**

**Supplemental Table S3.** Signal peptide proportions (with percentage) among protective antigens at different subcellular localizations.

|  | Adhesin/Total protective antigens count (percentage) | |
| --- | --- | --- |
|  | Gram-positive | Gram-negative |
| Extracellular | 17/36 (47.2%) | 8/33 (24.2%) |
| Cell Wall | 12/16 (75.0%) | - |
| Outer Membrane | - | 52/63 (82.5%) |
| Periplasm | - | 14/17 (82.4%) |
| Cytoplasmic Membrane | 2/9 (22.2%) | 2/10 (20.0%) |
| Cytoplasm | 1/10 (10.0%) | 3/41 (7.3%) |
| Unknown | 5/10 (50.0%) | 25/46 (54.3%) |
| Overall | 37/81 (45.7%) | 104/210 (49.5%) |

**Supplemental Table S4.** Adhesin distributions of different subcellular localizations among reported PAgs.

| Subcellular Location | Adhesin/Total protective antigens count (percentage) | |
| --- | --- | --- |
|  | Gram-positive | Gram-negative |
| Extracellular | 16/36 (44.4%) | 21/33 (63.6%) |
| Cell Wall | 14/16 (87.5%) | - |
| Outer Membrane | - | 52/63 (82.5%) |
| Periplasm | - | 9/17 (52.9%) |
| Cytoplasmic Membrane | 7/9 (77.8%) | 2/10 (20.0%) |
| Cytoplasm | 0/10 (0.0%) | 2/41 (4.9%) |
| Unknown | 9/10 (90.0%) | 25/46 (54.3%) |
| Overall | 46/81 (56.8%) | 111/210 (52.8%) |

**Supplemental Table S5.** Conserved domains computed for protective antigens and non-protective antigens using PfamScan.

**(See attached Excel file)**

**Supplemental Table S6.** Enrichment result of Clusters of Orthologous Group with adjusted p-value using EggNOG.

**(See attached Excel file)**

**Supplemental Table S7.** Enrichment result of Gene Ontology terms (Tab 1: Biological Process, Tab 2: Molecular Function, Tab 3: Cellular Component) with adjusted p-value using Argot2.

**(See attached Excel file)**

**Supplemental Table S8.** All bioinformatics programs used to compute peptide properties in this study.

| **Sequence-derived Features** | **Program** | **Download/Website Link** |
| --- | --- | --- |
| Subcellular localization | psortB^(1)^ | http://www.psort.org/psortb/ |
| Adhesin probability | SPAAN^(2)^ | https://sourceforge.net/projects/adhesin/files/SPAAN/ |
| Signal peptide | SignalP^(3)^ | http://www.cbs.dtu.dk/services/SignalP/ |
| Transmembrane alpha-helix | TMHMM^(4)^ | http://www.cbs.dtu.dk/services/TMHMM/ |
| Transmembrane beta-barrel | PROFtmb^(5)^ | https://rostlab.org/owiki/index.php/Packages |
| Conserved domain | PfamSCAN^(6)^ | http://ftp.ebi.ac.uk/pub/databases/Pfam/Tools/ |
| Gene Ontology | Argot2^(7)^ | http://www.medcomp.medicina.unipd.it/Argot2/index.php |
| Clusters of Orthologous Groups | eggNOG^(8)^ | http://eggnogdb.embl.de/#/app/home |
|  |  |  |
| (1) Yu, N. Y. et al. PSORTb 3.0: Improved protein subcellular localization prediction with refined localization subcategories and predictive capabilities for all prokaryotes. Bioinformatics 26, 1608–1615 (2010). | | |
| (2) Sachdeva, G., Kumar, K., Jain, P. & Ramachandran, S. SPAAN: A software program for prediction of adhesins and adhesin-like proteins using neural networks. Bioinformatics 21, 483–491 (2005). | | |
| (3) Petersen, T. N., Brunak, S., von Heijne, G. & Nielsen, H. SignalP 4.0: discriminating signal peptides from transmembrane regions. Nat. Methods 8, 785–786 (2011). | | |
| (4) Emanuelsson, O., Brunak, S., von Heijne, G. & Nielsen, H. Locating proteins in the cell using TargetP, SignalP and related tools. Nat. Protoc. 2, 953–971 (2007). | | |
| (5) Bigelow, H. R., Petrey, D. S., Liu, J., Przybylski, D. & Rost, B. Predicting transmembrane beta-barrels in proteomes. Nucleic Acids Res. 32, 2566–2577 (2004). | | |
| (6) Punta, M. et al. The Pfam protein families databases. Nucleic Acids Res 40 D290-D301. 30, 1–12 (2012). | | |
| (7) Falda, M. et al. Argot2: a large scale function prediction tool relying on semantic similarity of weighted Gene Ontology terms. BMC Bioinformatics 13, S14 (2012). | | |
| (8) Huerta-Cepas, J. et al. EGGNOG 4.5: A hierarchical orthology framework with improved functional annotations for eukaryotic, prokaryotic and viral sequences. Nucleic Acids Res. 44, D286–D293 (2016). | | |
